# Sulfur(VI) Fluoride Exchange (SuFEx)-Mediated Synthesis of the Chitosan-PEG Conjugate and Its Supramolecular Hydrogels for Protein Delivery

**DOI:** 10.3390/nano11020318

**Published:** 2021-01-27

**Authors:** Kyoung-Je Jang, Woong-Sup Lee, Sangbae Park, Jinsub Han, Jae Eun Kim, B. Moon Kim, Jong Hoon Chung

**Affiliations:** 1 Division of Agro-System Engineering, College of Agriculture and Life Science, Gyeongsang National University, Jinju 52828, Korea; kj_jang@gnu.ac.kr; 2 Institute of Agriculture & Life Science, Gyeongsang National University, Jinju 52828, Korea; 3 Department of Chemistry, College of Natural Sciences, Seoul National University, Seoul 08826, Korea; kabigon@snu.ac.kr; 4 Department of Biosystems & Biomaterials Science and Engineering, Seoul National University, Seoul 08826, Korea; sb92park@snu.ac.kr; 5 Department of Biosystems Engineering, Seoul National University, Seoul 08826, Korea; rhineop@snu.ac.kr (J.H.); je6740@snu.ac.kr (J.E.K.); 6 BK21 Global Smart Farm Educational Research Center, Seoul National University, Seoul 08826, Korea; 7 Research Institute of Agriculture and Life Sciences, Seoul National University, Seoul 08826, Korea

**Keywords:** supramolecular hydrogel, chitosan, sulfur(VI) fluoride exchange (SuFEx), protein delivery

## Abstract

Supramolecular hydrogels are considered promising drug carriers in the tissue engineering field due to their versatile nature. Chitosan hydrogels without chemical cross-linkers have low cytotoxicity and good delivery capacity; however, they have lower mechanical properties for injectable hydrogel usage. In this study, we developed novel chitosan derivatives via click chemistry for fabricating supramolecular hydrogels with higher mechanical strength under mild conditions. The chitosan derivative was successfully synthesized by a sulfur fluoride exchange reaction, and the synthesized chitosan-mPEG/Pluronic-F127 (CS-mPEG/F127) interacted with α-cyclodextrin (α-CD) to form a supramolecular hydrogel via a host-guest reaction. The gelation dynamics, hydrogel properties, and bovine serum albumin (BSA) release could be modulated by the concentration ratio of chitosan-mPEG and F127. This supramolecular hydrogel is a promising protein releasing carrier candidate for long term regeneration therapy.

## 1. Introduction

Hydrogels have recently attracted attention as an important material in the application of tissue engineering and regenerative medicine [1,2]. In particular, various natural/synthetic polymeric materials are used in the fabrication of hydrogels, have various properties depending on the type of polymer and the gelation method uses, and are applied to various fields accordingly [3]. Hydrogels have recently attracted increased attention in tissue engineering applications, such as soft tissue regeneration and in vitro three-dimensional cell culture [4,5]; in addition, hydrogels are widely used as materials for releasing drugs [6] or proteins [7]. Hydrogels can be classified by their crosslinking methods and their properties depending on the type of polymer and crosslinking method. Supramolecular hydrogels are one of the prominent candidates for tissue engineering applications. Supramolecular cross-linking has many noncovalent bonds, such as hydrogen bonds, metal-ligand coordination, host-guest recognition, and electrostatic forces with binding forces that are weaker than covalent bonds, limiting the mobility of the polymer at a macroscopic level and forming a three-dimensional network [8]. Conversely, chemically cross-linked hydrogels using cross-linkers have a permanent 3D structure and potential toxicity (due to the cross-linker used), which are disadvantageous to the loaded biomolecule stability of the hydrogels themselves.

Since 2014, Sharpless and coworkers have reported a sulfur(VI) fluoride exchange (SuFEx) reaction between a sulfonyl fluoride (R-SO_2_F) and an aryl silyl ether (Ar-OSiR_3_) or amine (R-NH_2_) under metal-free conditions [9]. SuFEx reactions have been continuously studied in bioconjugation chemistry [10,11] and drug discovery [12]. In the case of bio-SuFEx chemistries, amines are good counterparts of bio-SuFEx reactions and their conversion to sulfonamides by coupling sulfonyl fluoride with aliphatic amines [13,14]. The high stability of the sulfonamide linkage affords the driving force that makes this reaction a “click” process between two functional units. In addition, sulfonamides are well-known bioisosteres for amides, ureas, and carbamates in medicinal chemistry [15,16].

Alpha-cyclodextrin (α-CD) and PEGylated polymeric materials are representative materials that form supramolecular hydrogels based on host–guest reactions [17]. α-CD is a natural organic oligosaccharide with excellent biocompatibility, plays the role of host molecule, and has many advantages in its application in the biomedical field [18,19]. PEGylated molecules act as guest molecules, and PEGs(polyethylene glycols) and their decomposition products are known to be less toxic to cells, explaining the interest in their use as biomedical materials [20,21,22]. While the process of the host–guest reaction is not fast, there is an advantage of forming a gel even under mild reaction conditions. Hydrogels made through host–guest reaction are biocompatible compared to cross-linked hydrogels since they do not use toxic chemical cross-linker and are not exposed to UV rays. In addition, the gel structure transitions to a sol when shear forces are applied and then forms a gel again after a time [23]. Additionally, the thixotropic property of supramolecular hydrogels has an advantage in being used as a material for injections and 3D printing. In the research of Qianming Lin et al., a supramolecular hydrogel, which was physically crosslinked by mixing α-CD after synthesizing methoxy-polyethylene glycol conjugated with arginine-functionalized poly(L-lysine) dendron (MPEG-PLLD-Arg), was introduced. This hydrogel had a sustained drug release ability in the in vitro test and did not affect cell growth or viability. It was also used as a gene carrier in an animal model for cancer treatment; thus, it exhibited excellent biocompatibility and reduced the size of the tumor, confirming that it showed superior performance over PEI-25k [24]. In addition, supramolecular hydrogels hold great potential to be used for bioimaging. Supramolecular hydrogel-based bioimaging have greatly enhanced stability, reduced toxicity and improved target specificity compared to small-molecule-based bioimaging [25,26,27]. Therefore, we propose that hydrogels using novel PEGylated chitosan of a highly biocompatible polymer can be used as an effective carrier for proteins.

Chitosan is a polysaccharide-based polymer obtained by hydrolyzing chitin. Chitin can be obtained from insect and crustacean exoskeletons, fungi, and various kinds of natural substances. Chitosan is a biodegradable polymer and is attracting much interest in pharmaceutical and regenerative medicine because it has advantages such as nontoxicity, biodegradability, and wound healing ability [28]. In particular, as a carrier material for drug delivery and gene delivery systems, many studies have been conducted due to chitosan’s notable advantages [29]. However, chitosan hydrogels as drug/protein carriers have disadvantages compared to other delivery methods due to their relatively short release time and weak stability [7]. Many studies have been introduced to replace the functional moiety of chitosan with PEG to overcome its limits [30,31]. In particular, CS-g-PEG has been approved by the FDA as a material for in vivo insertion.

**Scheme 1:**
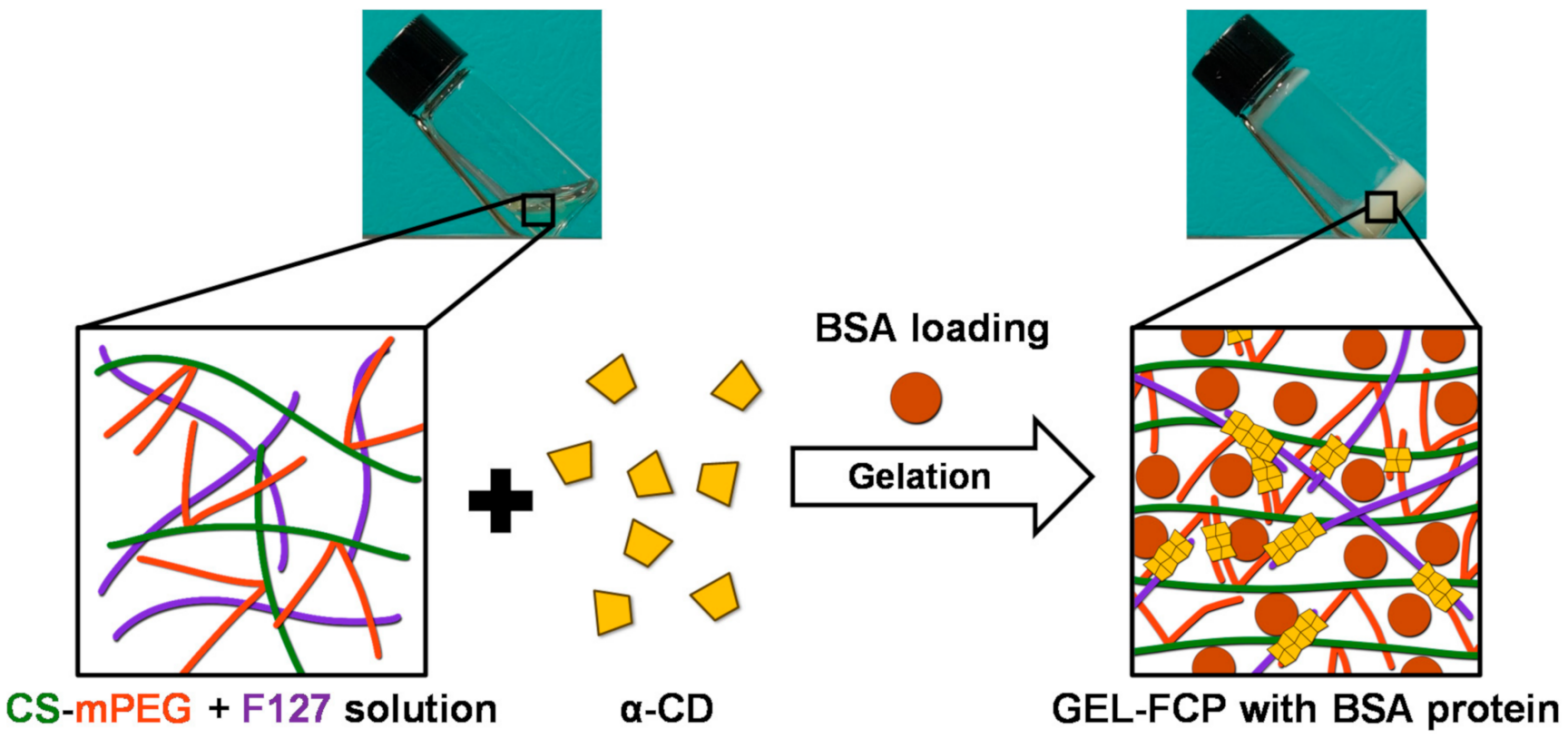
Schematic illustration of this study.

In this report, the guest molecule mPEG-chitosan was synthesized by sulfur(VI) fluoride exchange (SuFEx) reactions of chitosan-ESF and mPEG-NH_2_. The hydrogel was prepared using a host-guest reaction between CS-mPEG, F127(PEO-PPO-PEO), and α-CD (Scheme 1). According to the mixing ratio of CS-mPEG with F127, the hydrogel’s protein release characteristics were changed, and the release kinetics could thus be controlled. In addition, the mixing ratio of the two molecules also affected the hydrogel’s properties, and its characteristics were investigated by measuring the change in viscoelasticity. Additionally, whether the hydrogel can maintain its structure in the body was examined through in vitro experiments, and biocompatibility was confirmed through cytotoxicity evaluation.

## 2. Materials and Methods

### 2.1. Synthesis and Characterization of Chitosan Derivatives

All solvents and reagents were purchased from Sigma-Aldrich (St. Louis, MO, USA), Tokyo Chemical Industry Co. (Tokyo, Japan) or Samchun Chemicals (Seoul, Korea) and were used without further purification. Ethenesulfonyl fluoride was prepared according to the large-scale production method [32]. Methoxypolyethylene glycol amine was prepared according to the procedure [11]. The solid-state cross polarization magic angle spinning (CP/MAS) ^13^C and ^19^F NMR spectra of chitosan-derivative polymers were taken using Bruker Digital AVANCE™ III HD (500 MHz) NMR spectrometer (Billerica, MA, USA) at ambient temperature with a magic angle spinning rate of 7.0 kHz from National Center for Inter-University Research Facilities at Seoul National University.

### 2.2. Hydrogel Preparation

The F127 solution was prepared by dissolving it at a low temperature of 4 °C, while the α-CD solution with a 19.4% concentration was prepared by dissolving it in deionized water at no less than 70 °C. In addition, a CS-mPEG aqueous solution was prepared with deionized water at room temperature. First, the F127 solution and the CS-mPEG solution were mixed in proportion to each of the different types of solutions (0:1, 0.25:0.75, 0.5:0.5, 0.75:0.25, and 1:0), and the solution was vortexed for one hour to blend well with each other. The mixture of F127 and CS-mPEG solutions was then blended with an equal volume of α-CD solution, shaken briefly, and cultured at room temperature until the supramolecular hydrogel was formed. The formed hydrogels were named GEL-F, GEL-FCP-0.5, GEL-FCP-1, GEL-FCP-1.5, and GEL-CP. The formulation of the hydrogels is presented in Table 1.

### 2.3. Characterization of Chitosan Derivatives and Hydrogels

Fourier transform-infrared (FT-IR) spectra of chitosan and its derivatives (chitosan-ESF 1 and chitosan-PEG 2) were measured in the range from 4000–400 cm^−1^. FT-IR spectra were recorded on a PerkinElmer Spectrum Two spectrophotometer for solid and liquid samples. The internal structure of the hydrogels was observed using field-emission scanning electron microscopy (FE-SEM; SUPRA 55VP, Carl Zeiss, Oberkochen, Germany). The hydrogels were lyophilized and sputter-coated with platinum (approximately 20 nm thick). The observation was conducted at an accelerating voltage of 2 kV.

### 2.4. Gelation Time

The gelation times of the hydrogels with different mixing ratios were determined using the test tube inverting method. Briefly, 4 mL glass vials containing 1 mL of each hydrogel were dipped into a water bath at 37 °C. After the hydrogels became turbid, the fluidity of the hydrogels was observed every 30 s by gently tilting the vials. The time length when the hydrogels lost their fluidity was recorded. The measurement was repeated three times for each sample.

### 2.5. Rheology Study

For hydrogel characterization, gelation kinetics were analyzed through a fluidized time sweep by an advanced rheometric system in vibration mode with parallel plates (diameter 20 mm and 1.0-mm intervals) at 25 °C. The F127 aqueous solution, CS-mPEG aqueous solution, and mixed aqueous solution were mixed with α-CD solution to form a supramolecular hydrogel, and then an appropriate amount of specimen was placed on the plate and immediately measured. The mechanical properties of each hydrogel were analyzed at the same frequency. Oscillatory stress sweeps were also conducted to observe the viscoelastic behavior of the hydrogels under the condition that shear stress increases. The hydrogels were placed in 20 mm parallel plates and equilibrated at 37 °C until the gelation process was completed. The stress sweeps were performed at 1 Hz and 1 rad/s with oscillation from 10 to 1000 Pa. The storage modulus (G′), loss modulus (G″), and yield point were determined at the point where the hydrogel collapsed.

### 2.6. BSA Releasing

For the in vitro BSA release study, 200-μL samples of hydrogels containing 1% BSA were immersed in 1 mL of PBS (0.01 mol/L, pH = 7.4) at 37 °C. At predetermined time points, 100 μL of release medium was removed, and another 100 μL of fresh prewarmed PBS was added back to maintain the same total medium volume. Fifty microliters of released BSA was evaluated using a BCA protein assay kit (Sigma-Aldrich, St. Louis, MO, USA). All release studies were repeated five times. Cumulative BSA release was fitted by nonlinear regression analysis according to sigmoidal release model [33].

$$Q=A\left(1-{\;e}^{-k_{1}t}\right)+\;\frac{B}{1+{\;e}^{-k_{2}\left(t-T_{50}\right)}}$$

where *Q* stands for cumulative drug release, *t* is time, *A* is percentage of total drug release during phase I, *B* is percentage of total drug release during phase II, *T*_50_ is the time taken to release 50% of drug, *k*_1_ is the rate constant of drug release during phase I, *k*_2_ is the rate constant of drug release during phase II [34].

### 2.7. Cytotoxicity Assay

The mouse muscle cell line L929 (Korean Cell Line Bank, Seoul, Korea) were cultured in Dulbecco’s modified Eagle’s medium (DMEM; Welgene, Gyeongsan, Korea) supplemented with 10% (*v/v*) fetal bovine serum (FBS; Welgene, Gyeongsan, Korea) and 1% (*v/v*) penicillin-streptomycin antibiotic solution in a humidified 5% CO_2_ incubator. Cells were seeded in 96-well plates at 3 × 10^3^ cells/well and incubated for 24 h. Subsequently, cells were treated with growth medium containing different concentrations of F127 and CS-mPEG (2%, 0.2% and 0.02%). After 72 h, the cytotoxicity of the L929 cells was evaluated using s water-soluble tetrazolium salt assay kit (WST-1; Dogenbio, Seoul, Korea). The WST assay was performed according to the product instructions. After 1 h of incubation, the solution was transferred into a 96-well plate and analyzed at 450 nm with a microplate reader (Tecan, Männedorf, Switzerland).

## 3. Results

### 3.1. Synthesis of CS-mPEG Conjugate

**Figure 1:**
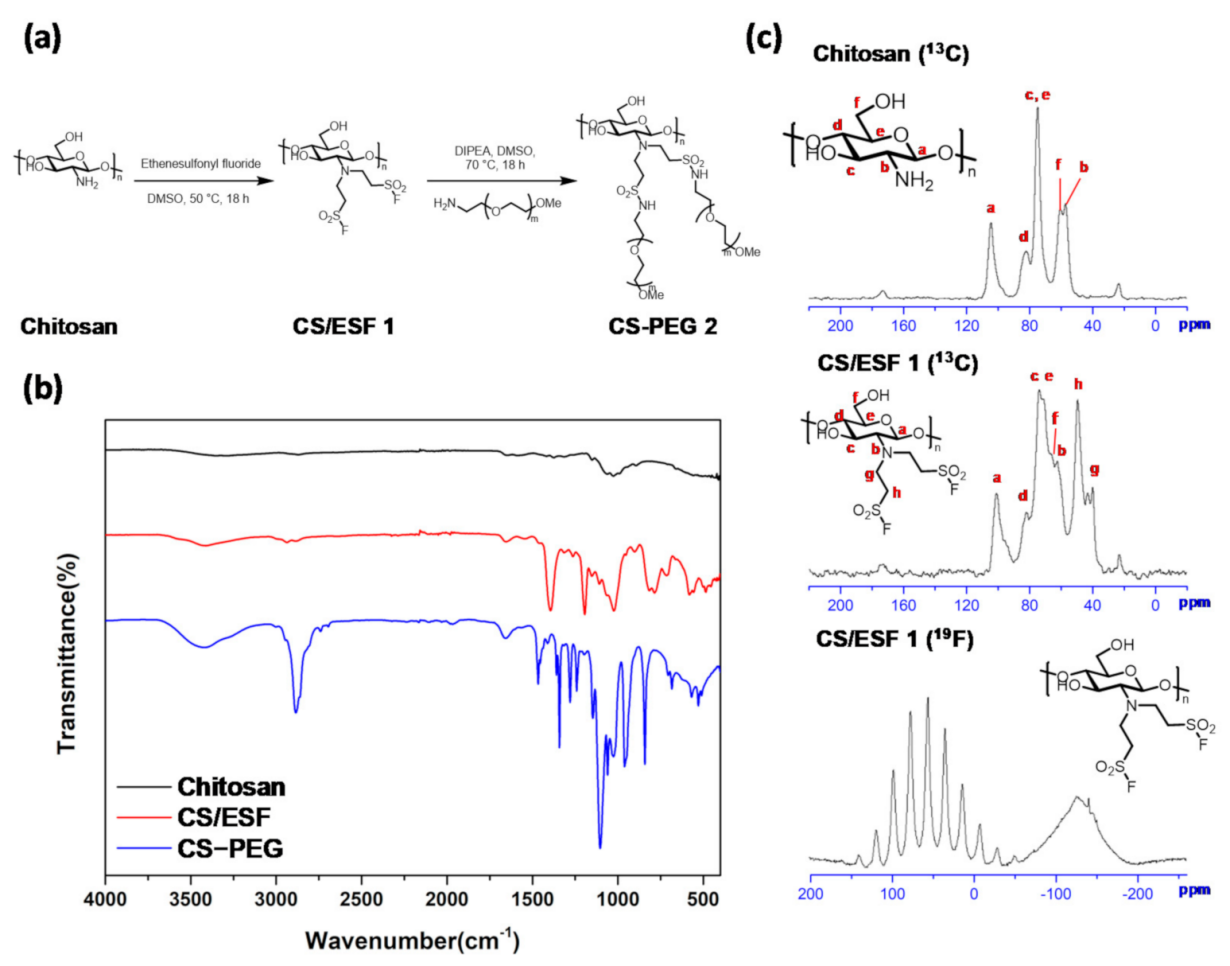
Synthesis and characterization of chitosan-PEG. (**a**) Synthetic route of CS/ESF 1 and CS/PEG 2. (**b**) FT-IR spectra of chitosan (black), CS/ESF 1 (red) and CS/PEG 2 (blue). (**c**) Solid-state NMR spectra of chitosan and CS/ESF 1.

To a well stirred mixture of commercially available chitosan (2.0 g, low MW, purchased from Sigma-Aldrich, St. Louis, MO, USA) in dimethyl sulfoxide (20 mL) was added ethenesulfonyl fluoride (ESF) (6.0 mL) in a round-bottomed flask equipped with a stirring bar. The mixture was stirred at 50 °C for 18 h. The solution was added slowly into a flask containing dichloromethane (100 mL) with vigorous stirring to precipitate the desired CS-ESF 1. The precipitate was dried under a high vacuum pump at 50 °C, to provide a pale-yellow powder (4.4 g, 98% yield), ^13^C NMR (125 MHz, CP/MAS) δ 173.3, 101.0, 82.0, 73.7, 71.2, 66.3, 62.2, 49.2, 42.6, 39.6, 22.8; ^19^F NMR (470 MHz, CP/MAS) δ 56.1 (Figure 1).

To a stirred solution of the precipitated CS-ESF 1 (0.70 g) in dimethyl sulfoxide (8.8 mL) were added *N*,*N*-diisopropylethylamine (3.5 mL) and methoxypolyethylene glycol amine (mPEG-NH_2_, MW 2000) (8.5 g). The reaction mixture was stirred at 70 °C for 18 h. The solution was added slowly into a flask containing methanol (100 mL) with vigorous stirring to precipitate the desired CS-mPEG 2. The precipitate was dried under a high vacuum pump at 50 °C to provide a brownish-yellow powder (7.3 g, 95% yield).

### 3.2. FT-IR Spectra for Chitosan and Chitosan Derivatives

The FT-IR spectra (Thermo Fisher Scientific, Waltham, MA, USA) of chitosan and its derivatives CS-ESF 1 and CS-mPEG 2 are recorded below (Figure 1b). The FT-IR spectra of chitosan reveal characteristic bands at 1650 cm^−1^, 1580 cm^−1^, and 1413 cm^−1^ (amide groups). The saccharine skeleton was characterized by absorption bands at 1150 cm^−1^ (asymmetric stretching of the C–O–C bridge) and 1070 cm^−1^ (C–O stretching). N–H and O–H stretching vibrations were characterized by broad bands in the region of 3500–3200 cm^−1^.

FT-IR spectra of CS-ESF 1 and CS-mPEG 2 exhibited additional stretches at 1398 cm^−1^, 1198 cm^−1^ (S=O), and 786 cm^−1^ (S–F). The characteristic stretching vibration of S–F of CS-ESF 1 at 786 cm^−1^ disappeared for CS-mPEG 2 with the appearance of a sulfonamide S–N stretching band at 684 cm^−1^, which indicates the SuFEx click attachment of mPEG-NH_2_ to chitosan-ESF.

### 3.3. Gelation Time

**Figure 2:**
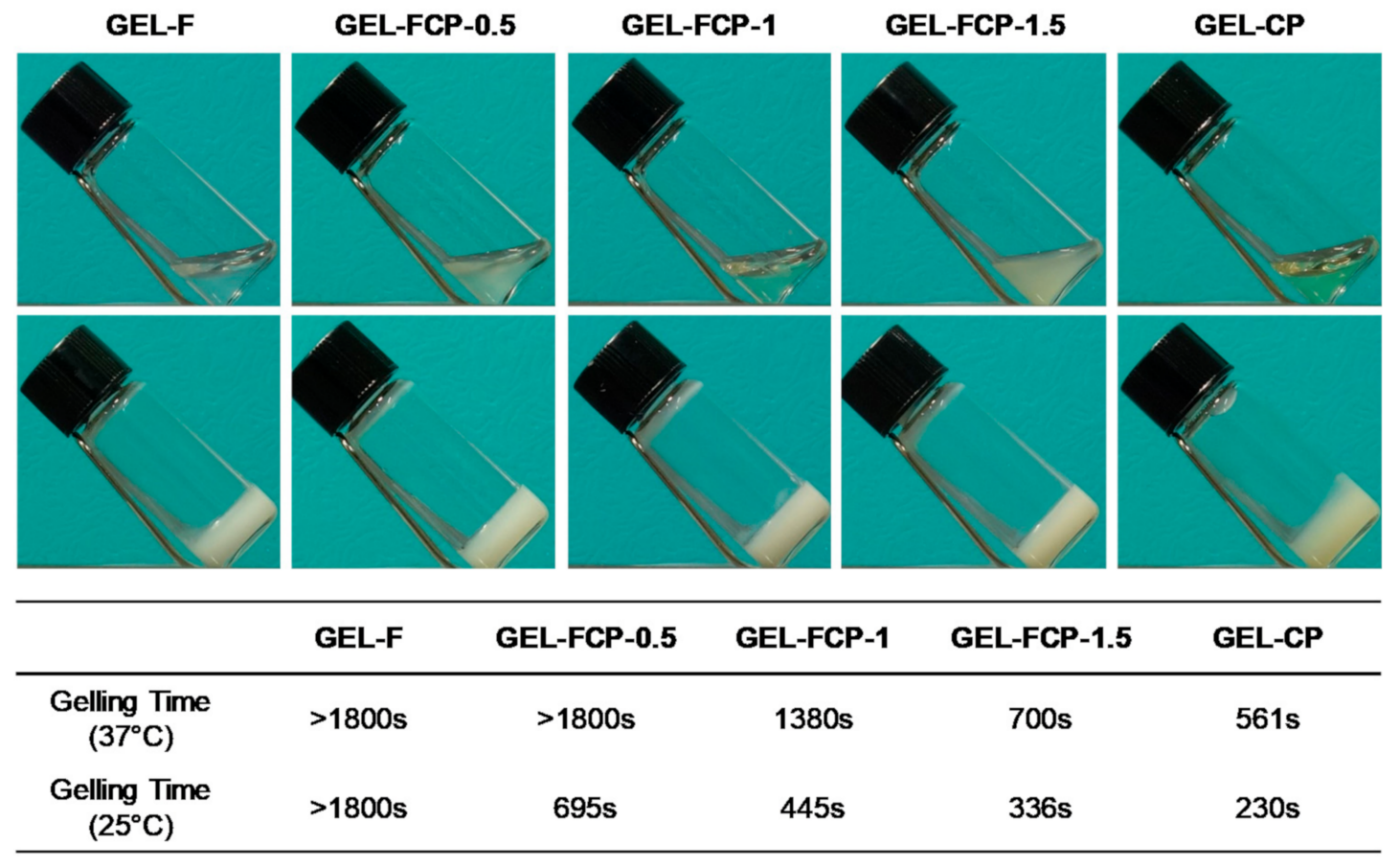
Photographs for the formation of a supramolecular hydrogel (in the mixed system between CS-mPEG and F127) of CS-mPEG/F127 and 7.0 wt% (in the mixed system) of α-CD at room temperature (25 °C) and body temperature (37 °C).

Single aqueous solutions of CS-mPEG could form yellowish transparent solutions at room temperature, and a single aqueous solution of F127 formed transparent solutions. After mixing aqueous solutions of F127 and CS-mPEG, yellowish transparent solutions were obtained. However, mixing the solutions with an α-CD aqueous solution with vortexing could form turbid yellowish mixtures (Figure 2). The gelation times of the mixture of CS-mPEG and the α-CD aqueous solution were measured at 25 °C (room temperature) and 36.5 °C (body temperature), and the results were measured at 230 s and 561 s, respectively. Under the same conditions, the gelation time for the mixture of F127 and α-CD aqueous solutions was measured to be more than 1800 s, and the exact gelation time was not estimated. However, the mixture could form a gel at room temperature overnight. The gelation times of the hydrogels consisting of F127, CS-mPEG, and α-CD solutions with various volume ratios (0.25:0.75:1, 0.5:0.5:1, 0.75:0.25:1) were also measured. The gelation time tended to increase as the ratio of F-127 increased.

### 3.4. Interior Structure

**Figure 3:**
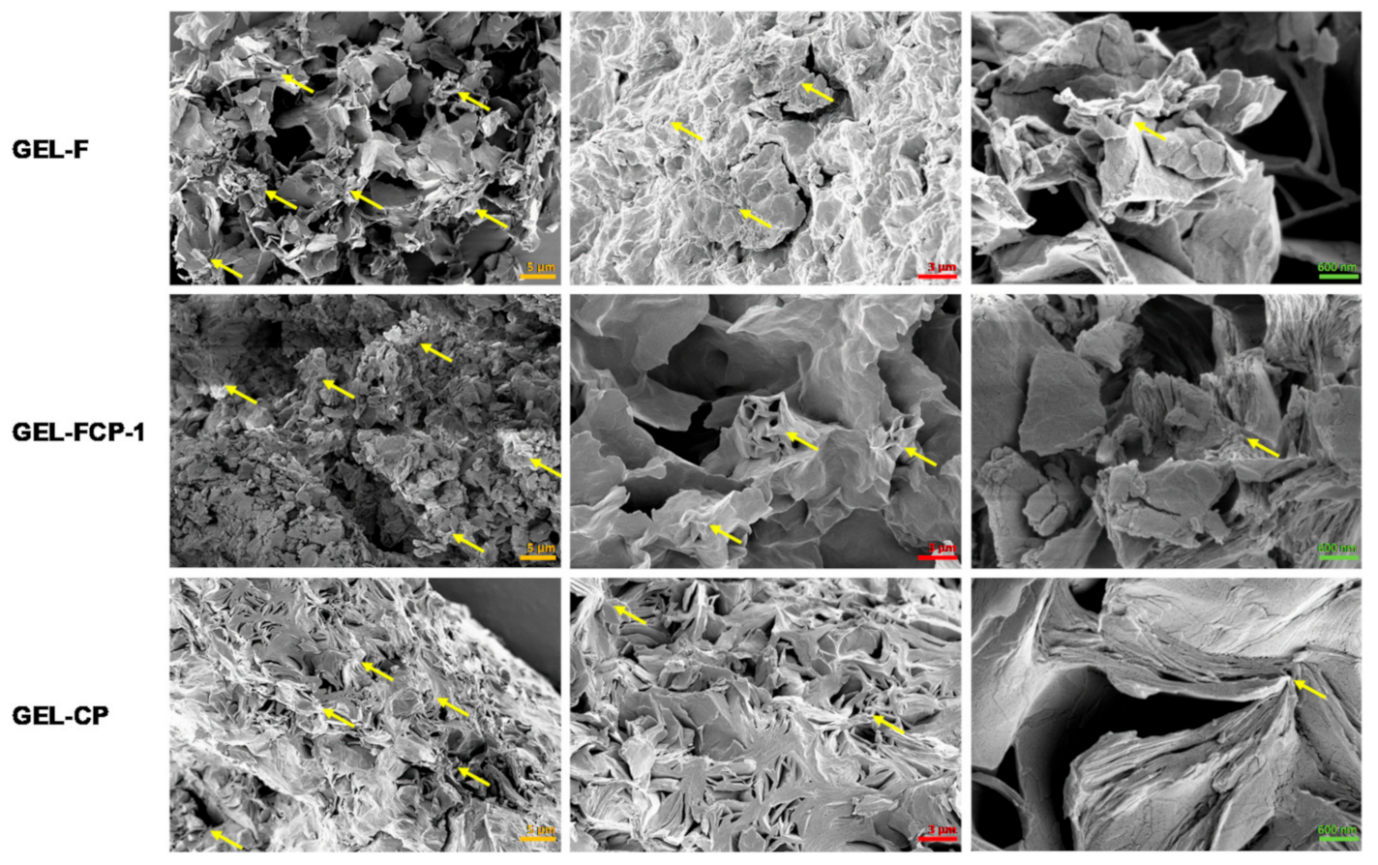
FE-SEM (field-emission scanning electron microscopy) micrographs of the microstructure of each samples. Yellow arrows indicate the aggregation zone consist of a firmed 3D structure formed by α-CD/mPEG and α-CD/PEO by host-guest reaction.

Field emission-scanning electron microscopy micrographs of freeze-dried F127/α-CD, CS-mPEG/α-CD, and CS-mPEG/F127/α-CD hydrogels were obtained. As shown in Figure 3, all hydrogels had stacked-like structures consisting of small pieces of plates. Among the stacked-like structures, some aggregation zones were found (yellow arrows). According to Peihong’s study [23], we could assume that the aggregation zone consists of a firmed 3D structure formed by α-CD/mPEG and α-CD/PEO by the host-guest reaction. In addition, we observed that GEL-CP has a more complex internal structure than GEL-F through the FE-SEM image.

### 3.5. Rheology Study

**Figure 4:**
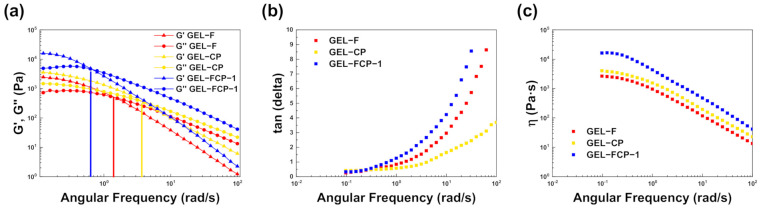
(**a**) G′ and G″ determined by oscillatory stress sweep measurements of each samples at the shear stress of 1.0 Pa. 200 s; (**b**) loss factor tan δ as a function of angular frequency; (**c**) shear viscosity of resultant each samples.

To investigate the effect of the mixing ratio between CS-mPEG and F127 on the rheological behavior, a time sweep measurement was carried out, and the storage modulus (G′) and loss modulus (G″) were observed by the angular frequency. Figure 4 shows the angular frequency-dependent profiles of G′ and G″ for samples formed by two types of polymer solutions, aqueous solutions of F127 and CS-mPEG with a 19.4% α-CD solution. All samples had a higher storage modulus (G′) than their loss modulus (G″), which means that they still have gel properties. The gel properties started to disrupt and changed into sol properties by increasing the ratio of the angular frequency. The F127- α-CD hydrogel had a lower sol-gel transition angular frequency point than the CS-mPEG-α-CD hydrogel.

The tangent delta function is used to indicate the ratio of values between the stored elasticity rate and the lost elasticity rate. As angular frequency increased for the F127 and CS-mPEG hydrogels, it was observed that the tangent delta value increased, and the rate of increase in tangent delta value was increased faster than that of the CS-mPEG hydrogel in the F127 hydrogel. Figure 4c shows the shear viscosity of F127 and CS-mPEG and α-CD supramolecular hydrogels. In both types of hydrogels, a tendency to decrease viscosity as angular frequency increased was observed. However, it can be seen that the viscosity of the CS-mPEG/α-CD hydrogel continues to be greater than that of the F127 α-CD hydrogel, even as the angular frequency increases.

**Figure 5:**
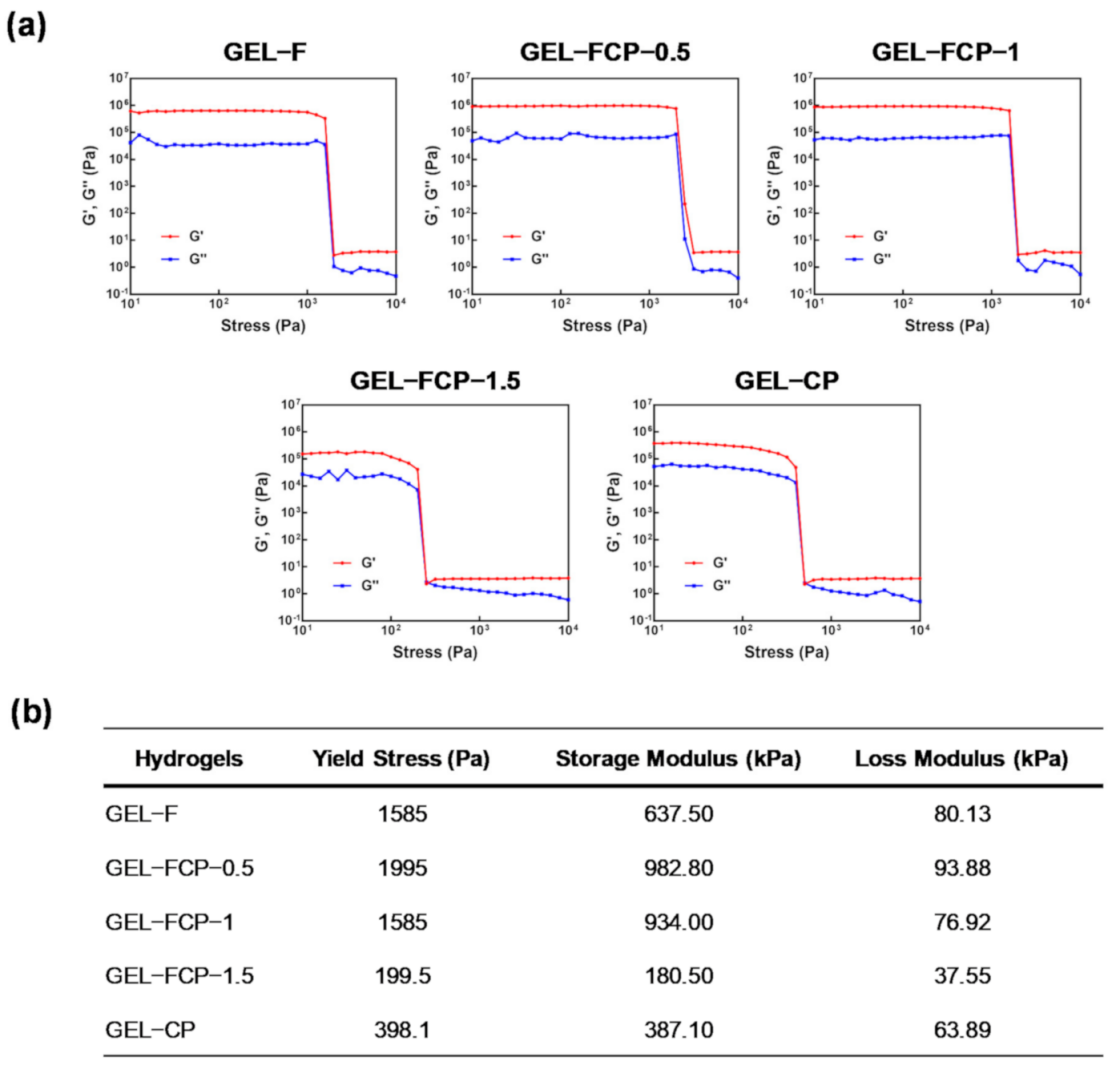
(**a**) G′ and G″ determined by oscillatory stress sweep measurements at the frequency of 1 Hz. (**b**) Yield stress, storage modulus (G′), and loss modulus (G″) of each samples.

The stress sweep analysis test was also conducted to measure the hydrogel’s elasticity and viscosity coefficient, which was fixed using a host-guest reaction using CS-mPEG, F127, and α-CD. This analysis was performed under two conditions: Measuring at 1 rad/s at angular velocity and holding the rotation ratio at 1 Hz. Figure 5 shows the measurement result of stress sweep under the condition of 1 rad/s. GEL-F and GEL-FCP-0.5 were not gelled and could not be measured. The yield point values of GEL-FCP-1, GEL-FCP-1.5, and GEL-CP were measured at 1000 pa, 158.5 pa, and 398.1 pa, respectively. The mean values and standard deviations of the yield stress storage, storage modulus, and loss modulus of each experimental group are presented in Figure 5b.

### 3.6. BSA Release

**Figure 6:**
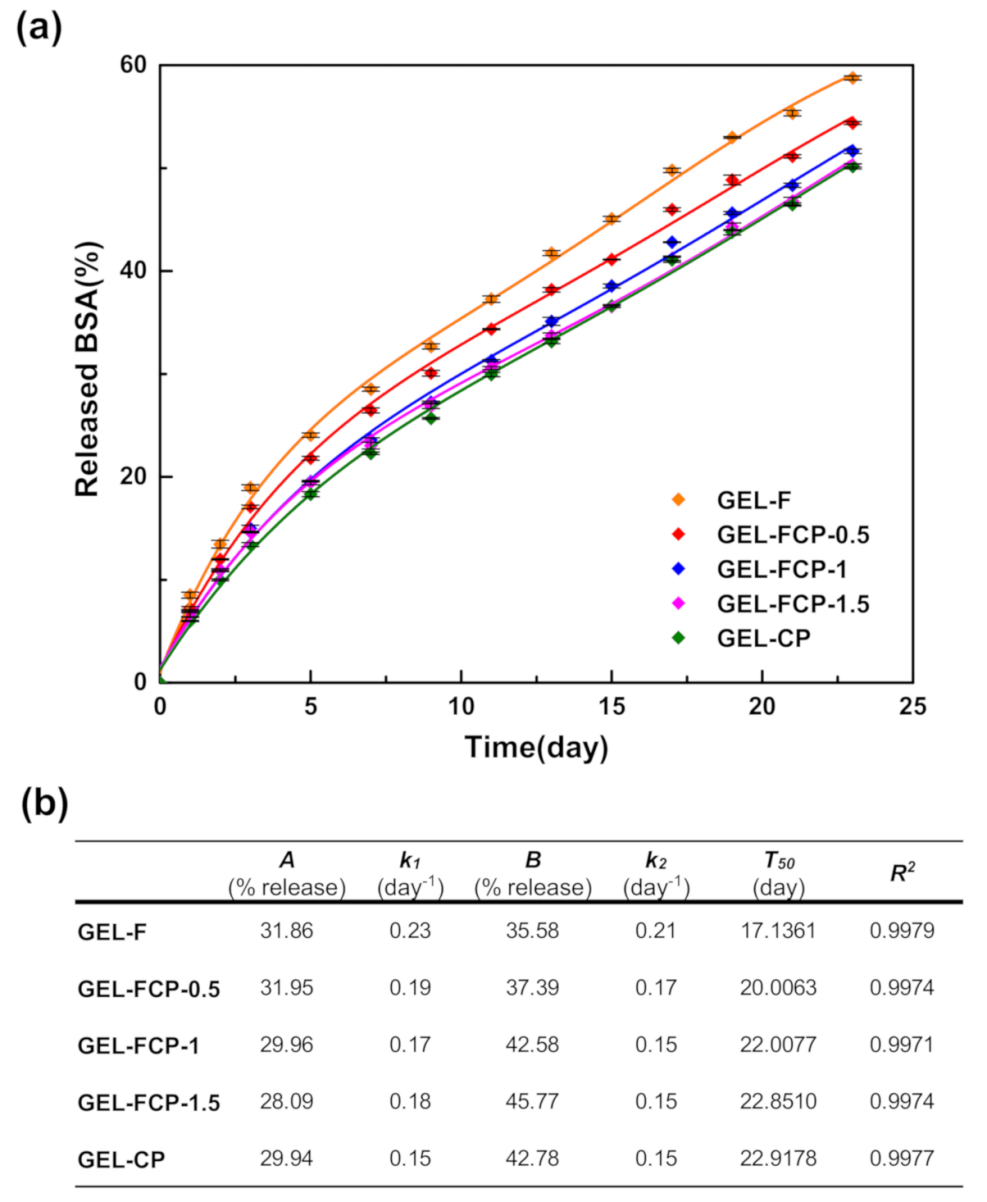
(**a**) Cumulative bovine serum albumin (BSA) release for 21 days (*n* = 5). The lines drawn represent nonlinear regression fit according to Equation (1). (**b**) Fitted parameters of each hydrogels.

The in vitro release profiles of BSA from hydrogels were studied using a Bradford protein assay. As shown in Figure 6a, BSA could be released from each supramolecular hydrogel above 21 days, and nearly all BSA could be released along with the degradation of the hydrogel matrix. The release rate of BSA from hydrogels was also affected by the mixing ratio between F127 and CS-mPEG. The GEL-F showed nearly 55.3% BSA release in 21 days, while the GEL-CP only showed nearly 46% BSA release. The hydrogels using the mixtures of F127 and CS-mPEG showed 51.1% (GEL-FCP-0.5), 46.3% (GEL-FCP-1), and 46.4% (GEL-FCP-1.5) BSA release. Fitted parameters were determined through nonlinear regression analysis according to equation 1 (Figure 6b). The drug release rate (*k*_1_ and *k*_2_) and the time taken to release 50% of drug (*T*_50_) showed a tendency to decrease as the content of CS-mPEG increased. Our results demonstrate that drug releasing profiles of the hydrogels can be controlled by the content of CS-mPEG.

### 3.7. Cytotoxicity

**Figure 7:**
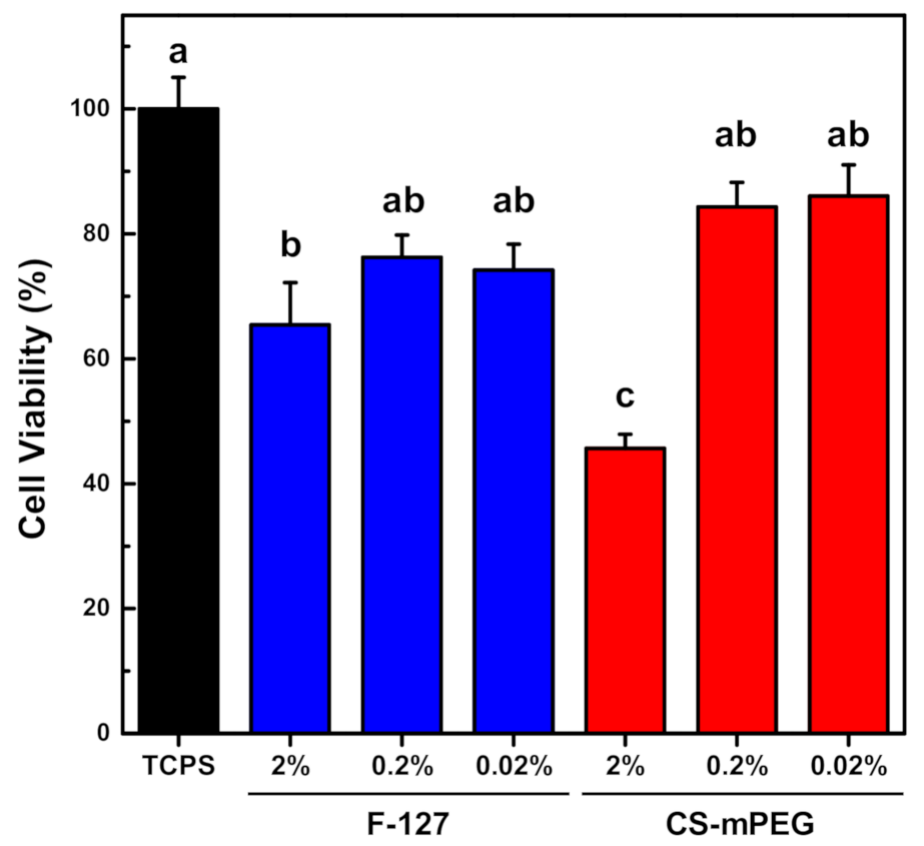
Viability of L929 cultured with growth medium with F127 and CS-mPEG. (ANOVA, Duncan’s multiple range test, *p* < 0.05). Same letters indicate that there is no significant difference between samples.

F127 and CS-mPEG materials were assessed for cell viability with the L929 cell (Figure 7). The cytotoxicities of the materials in the order F-127 and CS-mPEG were observed to be 2%, 0.2%, and 0.02%, respectively. The F127 and CS-mPEG with less than 0.2 wt% with proliferative medium showed no significant cytotoxicity.

## 4. Discussion

The use of a modified natural polymer for developing functional hydrogels is a critical technique due to its various advantages [35]. Notably, the biomolecule release properties, such as those for drugs, growth factors, and other biomolecules, from hydrogels have attracted much attention, and researchers are working to develop a novel hydrogel matrix to achieve effective release kinetics for drugs and to accomplish suitable injection for clinical use. Among a wide range of hydrogels, supramolecular hydrogels have shown promise in biological applications as drug delivery carriers, and various polymers have been tested for supramolecular hydrogels [24]. Chitosan, which is abundant in nature, has promise as a hydrogel material due to its biocompatibility and biodegradability. Some approaches to modify the functional groups of chitosan have been reported, and one of the chitosan derivatives, chitosan-g-PEG was approved by the FDA for excipient [36]. The purpose of this study was to develop novel hydrogels using chitosan derivatives as drug delivery carriers using click chemistry, to quantitatively investigate the rheological properties of supramolecular hydrogels and the cross-linking mechanisms and to evaluate its cytotoxicity.

To prepare the supramolecular hydrogel protein carrier, we used the mPEG and PEO segments for the guest molecules to interact with α-CD to form a hydrogel network. According to existing studies, mPEG and PEO segments are used as guest molecules to produce supramolecular hydrogels using α-CD. In most studies, hydrogel physical properties can be determined by the concentration of α-CD, which is due to the higher concentration of α-CD acting as a host molecule, increased entrapment with guest molecules, and more crosslinking sites. In particular, F127 is actively used as a temperature-sensitive hydrogel, but there has been interest in its high utilization as a host-guest reactive hydrogel using α-CD [23]. In our study, F127-α-CD was formed under conditions similar to existing research, and the release of BSA protein showed similar patterns. Additionally, CS-mPEG, which we developed, showed the successful formation of hydrogels using α-CD.

To determine gelation time, test tube inverting method was used. Gelation time of hydrogels tended to decrease as the content of CS-mPEG increased. This is because the host-guest reaction occurs more actively as the content of CS-mPEG, the guest molecules, increases. Interestingly, the gelation process of hydrogels was found to be slower at 37 °C than at 25 °C. This is attributed to increased mobility of α-CD. According to Wang’s study, α-CD would turn into a mobile sol phase when the temperature was increased above a certain temperature (Tgel−sol) [37]. To improve the physical properties of the hydrogel and the drug release dynamics, hydrogels mixing F127 and CS-mPEG were presented. Hydrogel drug-releasing kinetics and properties in the in vivo environment are known to be attributed to the properties and structure of the polymer that formed the hydrogel. Therefore, FE-SEM observation and analysis of XRD patterns were conducted to determine the mixing effects of the two types of polymers on the internal structure of hydrogel. The existence of the supramolecular CS-mPEG/α-CD and F127/α-CD inclusion structures was demonstrated by XRD (Figure S2). A shark peak appeared at 2*θ* = 19.9° in the XRD pattern in which α-CD threaded onto each mPEG and PEO segment in each polymer and then formed 3D physical crosslinks that induced the formation of the hydrogel by the host-guest reaction [23]. The appearance of plate materials arranged at regular intervals and flower-like shapes around specific points can be assessed to be similar to the internal structure common to supramolecular hydrogels. In particular, in GEL-F, the properties of the linear polymer F127 were reflected, and many plate stacking crystals were found compared to GEL-CP. GEL-CP has an internal structure that looks more random than that of GEL-CP. High-scaling images have found structures that appear to have built-up repeated structures, but have been assessed to be much less regular than GEL-F. This structure can be attributed to the lack of linearity and regularity in the chitosan structure. In addition, mixing the two polymers at a 1:1 mass ratio reduced the regularity of the pore and structure compared to GEL-F, but increased the shape compared to GEL-CP.

Our rheology studies have provided an interesting insight into how the properties of the internal structure affect the properties of the hydrogel. The fact that the storage modulus value of GEL-F, which showed the highest structural consistency, is higher than that of GEL-CP suggests that the hydrogel’s mechanical strength is also somewhat affected by the characteristics of the internal structure. The PPO-PEO-PPO structure of F127, a synthesized linear polymer, shows a much more regular structure than the molecular structure of irregular CS-mPEGs of natural origin. Therefore, it can be interpreted that the regular internal structure has a positive effect on the storage modulus value. We confirmed that in our hydrogel samples (GEL-CP, GEL-F, and GEL-FCP-1), G′ and G″s decrease as shear rate increases. The main reason for this phenomenon can be assumed to be the destruction of internal network structures by external energy. In addition, the similar tendency is also observed in other studies of the properties of the α -CD-based supramolecular hydrogel. Interestingly, it was found that the sol-gel transition point in the GEL-FCP mixed with two polymers was smaller than that of the point in the GEL-F and GEL-CP prepared with a single polymer. Furthermore, the shear viscosity of the GEL-FCP has been found to have consistently higher values than that of GEL-F and GEL-CP. From this phenomenon, we could assume that the bonding strength between homogeneous mPEG-α-CD (GEP-CP) or PEO-α-CD (GEL-CP) could be stronger than between mPEG-α-CD and PEO-α-CD (GEL-FCP). In addition, it was estimated that the shear viscosity at GEL-FCP-1 was the highest due to the entanglement of the polymer itself. The stress sweep test confirmed that GEL-CP endured much higher shear forces to maintain the structure of the hydrogel. These results may be attributed to the fact that the guest segment MPEG is firmly fixed to the chitosan backbone and that the distance of the guest segment within a molecule remains unchanged with external forces similar to the structure of a comb. However, it is located in the middle of the PEO linear polymer, and the guest segment of F127 is not fixed to molecules such as the chitosan backbone when external forces are applied, so the resistance to shear forces appears weak. The metaphysical results of the GEL-FCP experimental group can support this interpretation. In the case of GEL-FCP, we could see that the properties changed according to the ratio of chitosan-mPEG to F127. Most interestingly, we found that a certain percentage of GEL-FCP had a higher storage modulus value than those of GEL-F and GEL-CP. According to the literature, hydrogel formation can be controlled by the ratio of α-CD in the mixture. A higher concentration of α-CD could lead to accelerated inclusion interactions with mPEG and PEO segments and provide more physical crosslinking sites, which contribute to stronger and faster hydrogel formation.

Most of the physically crosslinked hydrogels and hydrogels we have developed undergo mass loss controlled by erosion through the decomposition of the bonds that build the network [38]. It is closely related to the release characteristics of the drug carrier and the decomposition of a hydrogel. Our in vitro experiments for observing the degradation of hydrogels have shown that mass loss does not occur rapidly, but continuously degrades at a steady rate, and CS-mPEG-α-CD has shown a higher level of stability than existing F127-α-CD (Figure S1). The reason for this phenomenon can be interpreted as the difference between the chitosan-MPEG molecule and the F127 molecular structure. The in vitro BSA release test results showed the characteristics of a continuous protein release profile for more than 21 days, similar to the degradation properties of the hydrogel. In particular, the BSA emission characteristics of the two types of hydrogels were found to be different, and it was confirmed that the BSA emission characteristics were controlled by mixing the two types of polymers.

The biocompatibility of carriers was also considered. Regardless of the cytotoxicity results in vitro, chitosan-mPEG/α-CD showed suitable biocompatibility for the drug delivery system according to the ISO 10993 experimental procedure. The main reason for the suitable biocompatibility is that the fabrication of supramolecular hydrogels does not include any toxic chemicals used in chemical bridging. The α-CD, PEO, and mPEG materials that make up the host and guest molecules of the supramolecular hydrogel are all biocompatible molecules. Therefore, the carriers we have developed can be injected into the body and can maintain function and stability for a long time.

## 5. Conclusions

In this study, to obtain an efficient and sustained protein carrier, we developed a novel CS-mPEG derivative via click chemistry. In addition, we suggested an injectable supramolecular hydrogel formed by our developed molecules with α-CD and F127 through host-guest interactions under mild conditions. The guest molecules CS-mPEG and F127 showed similar cytotoxicity. For rheology studies, the gelation time, mechanical strength, and shear viscosity of the obtained hydrogel could be modulated by a mixing ratio of CS-mPEG and F127. The results show that our material has acceptable injectability, and the degradation products have low cytotoxicity for biomedical applications. Our in vitro results suggested that this CS-mPEG/F127 supramolecular hydrogel could sustainably release the protein molecules. Considering the results presented in this study, GEL-FCP-1 is the most appropriate hydrogel since it exhibited adequate rheological properties and gelling time. Therefore, a CS-mPEG/F127/α-CD supramolecular hydrogel combined with a suitable biomolecule is considered to be a prominent candidate in long-term regeneration therapy.

## Figures and Tables

**Table 1:** Formulation of hydrogels.

Hydrogel	Polymers	Mass Ratio (wt%) F127:CS-mPEG	Mass Ratio (wt%) polymer:α-CD
GEL-F	F127	100:0	20:97
GEL-FCP-0.5	F127 + CS-mPEG	75:25	20:97
GEL-FCP-1	F127 + CS-mPEG	50:50	20:97
GEL-FCP-1.5	F127 + CS-mPEG	25:75	20:97
GEL-CP	CS-mPEG	0:100	20:97

## Data Availability

All the experimental data herein presented are available on request from the corresponding author.

## Supplementary Materials

The following are available online at https://www.mdpi.com/2079-4991/11/2/318/s1, Figure S1: Degradation of supramolecular hydrogels, Figure S2: XRD patterns (**a**) and crystal size (calculated by Scherrer’s equation) (**b**) of each sample.
