# Supplementary material for: Sulfur(VI) Fluoride Exchange (SuFEx)-Mediated Synthesis of the Chitosan-PEG Conjugate and Its Supramolecular Hydrogels for Protein Delivery
Source: Nanomaterials (Basel). 2021 Jan 27;11(2):318. doi: 10.3390/nano11020318 (PMC7912644; doi:10.3390/nano11020318)
Supplement: Supplementary file 1 [file nanomaterials-11-00318-s001.pdf]

Supplementary Materials

Sulfur(VI) Fluoride Exchange (SuFEx)-Mediated Synthesis of the Chitosan-PEG Conjugate and Its Supramolecular Hydrogels for Protein Delivery

Kyoung-Je Jang [1,2,†], Woong-Sup Lee [3,†], Sangbae Park [4,†], Jinsub Han [5,6], Jae Eun Kim [5], B. Moon Kim [3,*] and Jong Hoon Chung [5,6,7,*]

1 Division of Agro-System Engineering, College of Agriculture and Life Science, Gyeongsang National University, Jinju 52828, Korea; kj_jang@gnu.ac.kr
2 Institute of Agriculture & Life Science, Gyeongsang National University, Jinju 52828, Korea
3 Department of Chemistry, College of Natural Sciences, Seoul National University, Seoul 08826, Korea; kabigon@snu.ac.kr
4 Department of Biosystems & Biomaterials Science and Engineering, Seoul National University, Seoul 08826, Korea; sb92park@snu.ac.kr
5 Department of Biosystems Engineering, Seoul National University, Seoul 08826, Korea; rhineop@snu.ac.kr (J.H.); je6740@snu.ac.kr (J.E.K.)
6 BK21 Global Smart Farm Educational Research Center, Seoul National University, Seoul 08826, Korea
7 Research Institute of Agriculture and Life Sciences Seoul National University, Seoul 08826, Korea
* Correspondence: kimbm@snu.ac.kr (B.M.K.); jchung@snu.ac.kr (J.H.C.); Tel.: +82-2-880-6644 (B.M.K.); +82-2-880-4601 (J.H.C.)
† These authors equally contributed to this study.

Table of Contents

1.0 **General Remarks**

2.0 **Degradation of Supramolecular Hydrogels**

3.0 **XRD Spectra of Supramolecular Hydrogels**

1.0. General Remarks

All solvents and reagents were purchased from Sigma-Aldrich, Alfa Aesar, Tokyo Chemical Industry Co. or Samchun Chemicals and were used without further purification unless otherwise noted. Ethenesulfonyl fluoride was prepared according to the large-scale production method (K. B. Sharpless et al., J. Org. Chem., 2016, 81, 11360). Methoxypolyethylene glycol amine was prepared according to the procedure (B. M. Kim et al., Chem. Eur. J., 2018, 24 (43), 10948).

The solid-state cross polarization magic angle spinning (CP/MAS) 13C and 19F NMR spectra of chitosan-derivative polymers were taken using Bruker Digital Avance III HD (500 MHz) NMR spectrometer at ambient temperature with a magic angle spinning rate of 7.0 kHz from National Center for Inter-University Research Facilities at Seoul National University.

2.0. Degradation of Supramolecular Hydrogels

Degradation of supramolecular hydrogels was analyzed by measuring weight loss of the samples. 1000 μL of GEL-F, GEL-FCP-1 and GEL-CP was immersed in 500 μL of PBS and incubated at 37 °C. At predetermined time point, weight of the hydrogels was recorded after removing PBS. Then, 500 μL of prewarmed PBS was added back and incubated at 37 °C. Each sample was tested in triplicate.

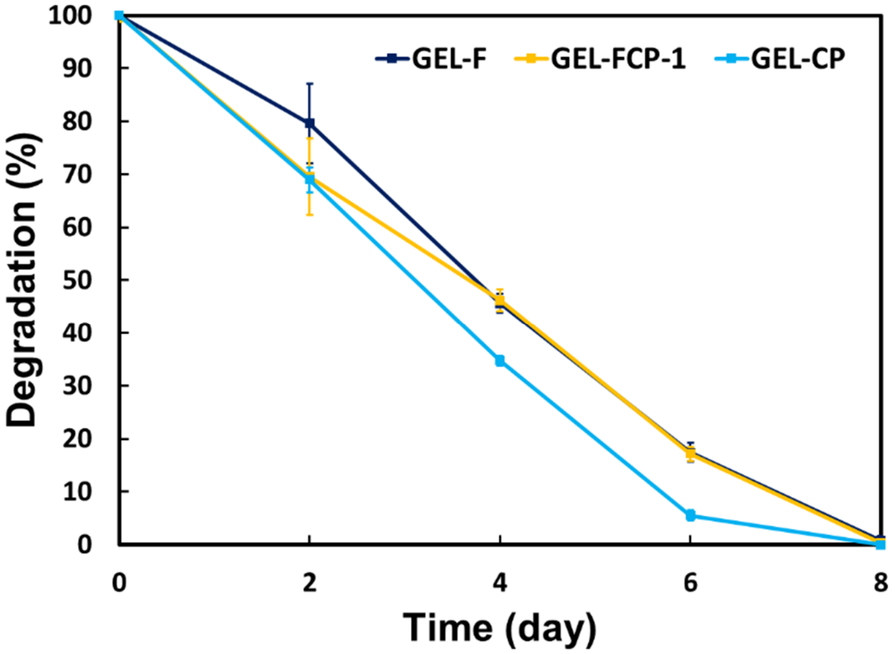

Figure S1. Degradation of supramolecular hydrogels.

3.0. XRD Spectra of Supramolecular Hydrogels

X-ray diffraction (XRD; D8 Advance, Bruker, Germany) was employed to investigate the crystalline phases of the hydrogels. Cu-Kα radiation was used for the measurements. The crystal sizes were estimated by the Scherrer equation.

a

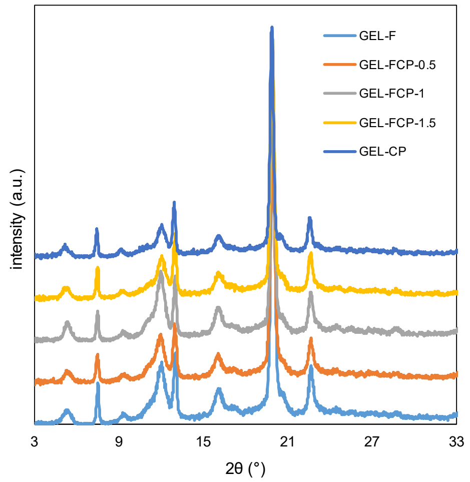

b

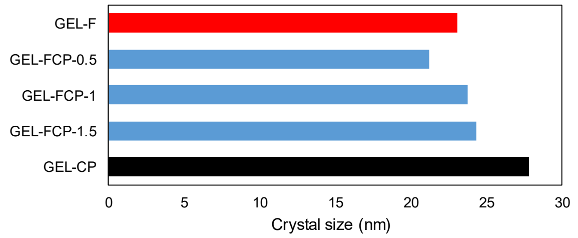

	2θ	Maximum intensity	FWHM	Dp (nm)
GEL-F	19.96	3661.718	0.36582	23.04
GEL-FCP-0.5	19.96	2862.030	0.39746	21.20
GEL-FCP-1	19.96	3093.578	0.35455	23.77
GEL-FCP-1.5	19.93	3095.228	0.34695	24.29
GEL-CP	19.91	2740.647	0.30348	27.77

Figure S2. XRD patterns (**a**) and crystal size (calculated by Scherrer's equation) (**b**) of each samples.

© 2021 by the authors. Licensee MDPI, Basel, Switzerland. This article is an open access article distributed under the terms and conditions of the Creative Commons Attribution (CC BY) license (http://creativecommons.org/licenses/by/4.0/).
